# Cost-effectiveness of a second opinion program on spine surgeries: an economic analysis

**DOI:** 10.1186/s12913-023-10405-x

**Published:** 2023-12-19

**Authors:** Eliane Antonioli, Daniel Tavares Malheiro, Vanessa Damazio Teich, Isabela Dias Paião, Miguel Cendoroglo Neto, Mario Lenza

**Affiliations:** Hospital Israelita Albert Einstein, Avenida Albert Einstein, 627/701 – Jardim Leonor – CEP, São Paulo, SP 05652-900 Brazil

**Keywords:** Low back pain, Neck pain, Spine Surgery, Quality of life, Cost-effectiveness, Orthopaedic Surgery, Second opinion

## Abstract

**Background:**

In this study we proposed a new strategy to measure cost-effectiveness of second opinion program on spine surgery, using as measure of effectiveness the minimal important change (MIC) in the quality of life reported by patients, including the satisfaction questionnaire regarding the treatment and direct medical costs.

**Methods:**

Retrospective analysis of patients with prior indication for spine surgery included in a second opinion program during May 2011 to May 2019. Treatment costs and outcomes were compared considering each patients’ recommended treatment before and after the second opinion. Costs were measured under the perspective of the hospital, including hospital stay, surgical room, physician and staff fees and other costs related to hospitalization when surgery was performed and physiotherapy or injection costs when a conservative treatment was recommended. Reoperation costs were also included. For comparison analysis, we used data based on our clinical practice, using data from patients who underwent the same type of surgical procedure as recommended by the first referral. The measure of effectiveness was the percentage of patients who achieved the MIC in quality of life measured by the EQ-5D-3 L 2 years after starting treatment. An incremental cost-effectiveness ratio (ICER) was calculated.

**Results:**

Based upon the assessment of 1,088 patients that completed the entire second opinion process, conservative management was recommended for 662 (60.8%) patients; 49 (4.5%) were recommended to injection and 377 (34.7%) to surgery. Complex spine surgery, as arthrodesis, was recommended by second opinion in only 3.7% of cases. The program resulted in financial savings of -$6,705 per patient associated with appropriate treatment indication, with an incremental effectiveness of 0.077 patients achieving MIC when compared to the first referral, resulting in an ICER of $-87,066 per additional patient achieving the MIC, ranging between $-273,016 and $-41,832.

**Conclusion:**

After 2 years of treatment, the second opinion program demonstrated the potential for cost-offsets associated with improved quality of life.

**Supplementary Information:**

The online version contains supplementary material available at 10.1186/s12913-023-10405-x.

## Background

Back pain is the most common cause of musculoskeletal disability worldwide [1–4]. In the last decade, the mean inflation-adjusted annual expenditures on medical care for patients with Spine Related Disorders (SRD’s) increased by 95%, with more than $100 billion spent annually on these disorders [5, 6]. A study about the US spending on personal health from 1996 to 2013 demonstrated that low back and neck pain accounted for the highest amounts of spending [7]. In 2016, among the 154 conditions, low back and neck pain had the highest amount of health care spending with an estimated $134.5 billion [8]. Whether it is neck or low back, spinal fusion is one of the top ten surgical procedures performed in the United States of America [9, 10]. Additionally, SRDs are the leading cause of years lived with disability (YLDs) and one of the main reasons for activity limitation and work absence [11, 12]. Needless to say, SRDs are a growing global public health concern.

Surgical intervention for SRDs is indicated only if symptoms persist despite initial conservative management and/or if progressive neurologic deficit occurs [3, 13]. Unfortunately, the uptake of current clinical practice guidelines is “weak” at best with 52% of physicians following current practice guidelines and, in some cases, as few as 10% of patients receiving evidence-based care [13].

However, there is no consensus regarding the best type of surgery to treat those conditions and several options exist for the treatment of degenerative disease of the spine. Different surgical modalities with a wide variation in complexity are used for similar diagnoses [14–16]. In the last 2 decades, there has been a substantial increase in the frequency and complexity of surgical procedures used to treat back pain. This increase has been correlated with a rise in overall healthcare costs and utilization [8, 17–21]. However, there is no agreement on any incremental cost-effectiveness improvements that may be related to the use of new techniques [22, 23].

Therefore, in order to provide the best intervention for patients and reduce unnecessary care, efforts towards quality improvement in spine surgeries should consider the economic aspects of intervention and focus on the technical quality of the care provided [19]. Cost-effectiveness studies concerning interventions for low back pain have been conducted worldwide [24–26] and a recent systematic review pointed out the need for more economic evaluations comparing exercise therapy with drugs and surgery for patients with neck and low back pain [27].

In response to this scenario, the Hospital Israelita Albert Einstein (HIAE) developed a multidisciplinary second opinion program to evaluate patients referred for surgical treatment for degenerative spinal disease. This program, entitled “Second Opinion on Spine Surgeries”, is an attempt to assist the medical decision making associated with these conditions [28] inside the context of the Brazilian private healthcare system, where medical doctors are reimbursed by the insurers and direct costs are provided by the healthcare institution [29].

Second opinions for spine surgery have been considered as a potential strategy to improve surgical decision making and patient outcomes [28, 30]. The topic is relevant, and two scoping reviews on second opinion for spine surgery has recently been published, investigating the frequency and impact of second opinions between studies [31] and, comparing the concordance rates in diagnoses and type of surgery, number of surgeries, patient-reported outcomes, costs, and health care use associated with second opinion programs [32]. These reviews concluded that there is a need for a study comparing clinical outcomes between those who received versus did not receive a second opinion and this potential for improving surgical decision-making, financial costs of spine surgery and patient outcomes.

So, in this study we proposed a new strategy to measure cost-effectiveness. We used the minimal important change (MIC) in the quality of life reported by patients as clinical endpoint. This includes assessing the satisfaction questionnaire regarding the treatment after 2-year period. Our strategy highlights the patient’s perception of their treatment and considers the significant change in quality of life that matters to the patient and changes experienced by patient over time. For comparison analysis, we analyzed data from our clinical practice. We specifically looked at data from patients who underwent the same type of surgical procedure as recommended by the first referral. The objective of this study was to assess the cost-effectiveness of a program that provides a second opinion on spine surgery. We measured effectiveness by examining the improvement in patients’ quality of life (Utility) as a measure of effectiveness, and direct medical costs under the perspective of a Brazilian private hospital.

## Materials and methods

### Study design and population

This was a retrospective observational study of patients who took part in the Second Opinion on Spine Surgery Program, between May 2011 and May 2019, at HIAE, a private not-for-profit philanthropic hospital with 711 beds in the city of São Paulo, Brazil. HIAE is a tertiary hospital with access to private health coverage.

The inclusion criteria for this study were: patients to whom the initial recommended treatment was spine surgery (neck or back) by a spine surgeon not participating in the program; adults of both sexes older than 18 years with no medical contraindication to general anesthesia; who understood Portuguese; and patients who had completed 2 years of follow-up of clinical outcomes after initiating treatment for spine condition. Exclusion criteria were people with spinal fractures, major scoliosis, congenital spinal deformities or infection. Information was retrospectively gathered from the HIAE database; the Hospital Israelita Albert Einstein Research Ethics Board approved the study (number: 37340820.6.0000.0071).

### Data sources and study variables

This study was developed using the same structure and setting of the HIAE Spine Center described in a previous publication [33]. Patients with previous indication of surgery for degenerative spine diseases were referred to the Spine Center by their healthcare provider. As soon as the patient contacted the Spine Center, he or she was offered an opportunity to obtain a second opinion on their spine health condition. If they consented, they were evaluated by a clinical spine specialist, physiatrist, or orthopedist that did not have any financial or other benefit, from any of the recommended treatments or outcomes.

Initial medical evaluation consisted of medical history followed by physical examination and a review of all related records. When necessary, additional medical imaging or laboratory investigations were requested. Upon completing the evaluation, the physician from the second opinion program, who is not a spine surgeon, could recommend conservative management or surgical intervention.

When there was consensus that conservative management was recommended rather than surgery, appointments were offered to patients and, if they accepted, the patient was referred to a rehabilitation center inside the Spine Center. Patients received an evaluation from a physiotherapist and based on their presentation, could receive manual therapy, exercise therapy, hydrotherapy and/or acupuncture. Of the patients recommended conservative treatment in the second opinion, 75% stated they had received physical therapy previously. Additionally, 80% of these patients stated that they were not engaging in any physical activity. All participants who received conservative management were reevaluated by physicians after 10 sessions. If their progress was satisfactory (related improvement in pain and function), they could be discharged with home exercises or continue conservative management for 10 more sessions. If they had not improved or worsened, they were referred to a spine surgeon from the HIAE Spine Center.

When there was consensus that surgery was recommended or when consensus could not be reached on the initial evaluation, patients were randomly sent to one of the specialists in spine surgery. The surgical staff is formed by nine teams of spine surgeons, four led by a head neurosurgeon and five by an orthopedist spine surgeon, each having more than 15 years of spine surgery experience. After the consultation with the surgical team, the same or a different type of surgery could be recommended. The surgery indication needed to follow an evidence-based protocol established and approved by the specialists’ members of the multidisciplinary Spine Center team (physicians, nurses and physical therapists). Members of the Spine Center multidisciplinary team attend this “spine board” under the supervision of the hospital orthopedic management team. The aims of the spine board were: (1) to discuss the technique in surgical cases and (2) to reach a consensus recommendation for conservative management or surgery. If surgery was recommended, the procedure was performed by one of the nine surgeons on the spine board, chosen at random.

All participants who received a second opinion and had a previous recommendation for surgery from the first referral, were given the option to receive treatment at HIAE. They could agree or disagree with the second opinion recommendation and proceed with the procedure of their choice. If participants declined to consult with the spinal board, the final diagnosis was recorded after the consensus meeting between the physiatrist and orthopedic surgeon. However, no final treatment plan was recorded or planned in these cases.

### Clinical endpoint

All participants initially met with a senior nurse, member of the Spine Center, who explained the second opinion process. The nurse recorded the first referral diagnosis, collected demographic data and asked each participant to complete the EuroQol-5D-3 L (EQ-5D-3 L) questionnaire, validated to Portuguese language, which measures perceived health in five dimensions [34]. Each participant’s score was transformed into a Utility score using established preferences based on the Brazilian population [35]. Utility values closer to 1 represent better health states and scores closer to 0 represent health states nearer to death [36].

All patients undergoing spinal surgery at HIAE are followed up by the outcomes department, which collects demographic data, clinical endpoints and patient reported outcomes. This clinical outcome was collected on the initial assessment and 2 years after treatment, by telephone.

Patient-reported outcome measures (PROMs) related to physical functioning were also collected at baseline and 2 years after treatment. However, different instruments and metrics were applied to patients with neck and back disease, not allowing comparability between instruments in the study population. Therefore, these outcomes were not included in the analyses.

In order to estimate the costs and benefits related to the first referral, real data from patients who underwent the same type of surgical procedure recommended by the first referral at HIAE, with a 2-year follow-up after the procedure, were used as a proxy. For this, through a search in the institutional database, patients who underwent the same surgery recommended in the second opinion program were located. Their costs and variations in Utility were used as the estimated achievable result in case the first referral procedure had been performed. Patient selection for this estimation was controlled for sex, age, disease and Utility pre-intervention to try to estimate more accurately the costs and outcomes expected for those patients.

### Economic assessment

A cost-effectiveness analysis was developed, in order to calculate the incremental cost per additional patient achieving the MIC when comparing the second opinion to the first referral in a 2 year follow-up (as defined below), considering all treatment costs related to the clinical condition studied during the period, treatment failures and reinterventions, as well as the measure of Utility gain during the period. The analysis was completed using the perspective of a tertiary level private hospital.

The costs included direct medical costs, considering outpatient conservative treatments and in-hospital costs related to surgery and treatment of complications, extracted through the hospital management system (Intersystem Trackcare®). Medical fees were estimated considering the average fee schedule negotiated with payers in 2021. The hospital costing methodology considers the apportionment of the labor force used in the hospital care process including fees and charges, based on the average time and number of professionals required for the activity. The costs were converted from Reais (Brazilian currency) to US dollars and adjusted to the cost schedule of June 2021 (https://economia.acspservicos.com.br/indicadores_iegv/iegv_dolar.html, i.e. 1 Real = US 5.0313), in order to avoid that the effect of inflation on the medical inputs influences the analysis [37]. The conversion of monetary values into terms of GDP per capita was utilized for international comparison purposes. In 2021, the Net Benefits, based on a willingness to pay of 1 per capita GDP, were $8,396.94.

The effectiveness was calculated as the percentage of patients considered as responders, for having achieved the MIC in 2 years, based on the difference in quality of life (Utility) measured through the EQ-5D-3 L, i.e., as a minimum gain of 0.2571 points in the Utility.

### MIC estimation

The MIC was established as treatment efficacy, which refers to the smallest change in Utility that patients consider important [38, 39]. The anchor-based method was performed to estimate the MIC for the current analysis, which uses a single question at follow-up as the external criterion, defined as anchor question, asking patients how much they have changed [39]. The anchor question used was: ‘How satisfied are you with the results of your treatment?’. The respondent can grade as: ‘Very satisfied’, ‘Satisfied’, ‘Neither satisfied nor dissatisfied’, ‘Dissatisfied’ and ‘Very dissatisfied’. From the anchor question, the states of ‘Very satisfied’ and ‘Satisfied’ were defined as success and graded with a value of 1. The other answers were indicated as failure and graded as 0. The MIC is defined as a threshold in quality of life (Utility), using the formula defined by Terluin et al [39]:

$${\rm{MIC}}\,{\rm{ = }}\,{\rm{gMIC}}\,{\rm{ + }}\,{\rm{S}}\,{\rm{x}}\,{\rm{log}}\,{\rm{-}}\,{\rm{odds}}\,\left( {{\rm{ratio}}} \right)$$

gMIC = (“genuineMIC”), which ranged from 2.9 to 23.4. When the proportion of improved patients is 0.5, we have MIC = gMIC.

ratio = proportion of patients with improvement / 1-(proportion of patients with improvement).

S = (slope coefficient) 0.090 × SD + 0.103 × SD × Cor (represents the standard deviation of the observed change score).

where:

SD = standard deviation of observed change score.

Cor = correlation between the observed change score and anchor question.

In order to avoid overestimation or underestimation in defining the predicted MIC, the adjusted MIC analysis was added to the formula, as suggested by Terwee et al. [38]. Predictive modeling of adjusted MIC is indicated when the proportion of improvement of patients is greater than 50%. Adjustment is performed using log-odds of improvement, based on the standard deviation of the Utility change and the correlation between the Utility change and the anchor question. Thus, resulting in an adjusted MIC independent of the proportion of improvement of the patients analyzed. The adjusted MIC was calculated using the formula:

$$\begin{array}{l}{\rm{MI}}{{\rm{C}}_{{\rm{adjusted}}}}{\rm{ = MI}}{{\rm{C}}_{{\rm{predicted}}}}{\rm{- }}\left( {{\rm{0}}{\rm{.0090 + 0}}{\rm{.103 x Cor}}} \right){\rm{ }}\\\,\,\,\,\,\,\,\,{\rm{x S}}{{\rm{D}}_{{\rm{change}}}}{\rm{x log - odds }}\left( {{\rm{ratio}}} \right)\end{array}$$

Where:

MIC_adjusted_ = adjusted minimal important change;

MIC_predicted_ = adjusted for the proportion improved patients;

Cor = correlation between the Utility change and the anchor question;

SD_change_ = standard deviation of the Utility change;

### Adjusted MIC analysis

The MIC reference was defined using data from patients who underwent surgical intervention at HIAE, as recommended by the second opinion program and that completed follow-up in 2 years, including the satisfaction questionnaire regarding the treatment (the anchor question). The analysis excluded patients who received conservative treatment to avoid any confounding effects, as second opinion program implemented a different approach. Out of the total of 365 surgical patients, 279 met the criteria for MIC analysis, representing 76.4% of the total., These patients provided data on their quality of life and satisfaction with the treatment results. A sample size analysis was conducted to determine the minimum number of cases needed, considering a sampling error of 5% and a confidence level of 95%. And it was determined that ae minimum of 188 cases is required, considering the distribution of the most heterogeneous population (worst case). So, the MIC reference was then calculated as a minimum gain of 0.2571 points in the Utility measure when comparing 2 years after the intervention versus baseline, in order that patients consider the treatment satisfactory. The data used in the estimated MIC analysis are available in the supplementary file (Additional Table 1).

### Costs and utilities estimation

The costs and utilities of the second opinion procedures performed at HIAE were measured using actual utilization and costs at the hospital, as well as the outcomes reported by patients. To facilitate comparative analysis, these parameters had to be estimated for the procedures recommended by the first referral. This estimate data was generated through based on our clinical practice and data from 377 patients who underwent the same type of surgical procedure as recommended by the first referral, between May 2011 and May 2019 at HIAE. The patients were paired accordingly based on their type of procedure recommended in the first referral with surgical patients who had undergone the same procedure at the hospital. This allowed us to estimate the average cost and average Utility gain per surgical indication. The hospital costs and medical fees were based on the June 2021 fee schedule. The outcome was evaluated by the percentage of patients who experienced clinical improvement, which was determined by comparing their Utility score between 2 years and baseline, ensuring that the improvement was at least the MIC Thus, we estimated costs and utilities, that would have been achieved had the 1088 surgical indications in the first referral treatment been performed. We obtained this information from analyzing data collected from patients with the same diagnosis who underwent the same surgery recommended by the first referral at HIAE and had a 2-year follow-up. To estimate these costs and utilities, we used the average cost and outcome values of the group undergoing the same surgical intervention within the second opinion program.

Complication rates for second opinion procedures were recorded based on chart review and were estimated for patients from the first referral. This estimation was made considering the same rates observed for these procedures when performed at HIAE. The cost of the complication was estimated as the same cost of the initial referral, considering the need for reoperation, performing the same procedure as the first intervention. The estimated Utility values for each of the first referral groups is shown in Table 1. Regarding the conservative treatment recommended by the second opinion, the probability of patients needing surgical intervention after treatment was also calculated, as well as the respective costs.

With this strategy, it was possible to compare, at the individual level, the actual cost and outcomes results of the second opinion program and estimated costs and outcomes of patients with the same surgical indication by the first referral performed in the hospital (database).

**Table 1:** Estimated utilities and costs related to first referral treatment in 24 months (N = 1,088)

FIRST REFERRAL	ESTIMATED UTILITY	ESTIMATED COST
Arthrodesis	0.74	$10,064.07
Decompression	0.58	$7,019.12
Hernia	0.95	$6,350.88
Injection	0.69	$3,287.14
Rhizotomy	0.53	$2,034.27

**Fig. 1:**
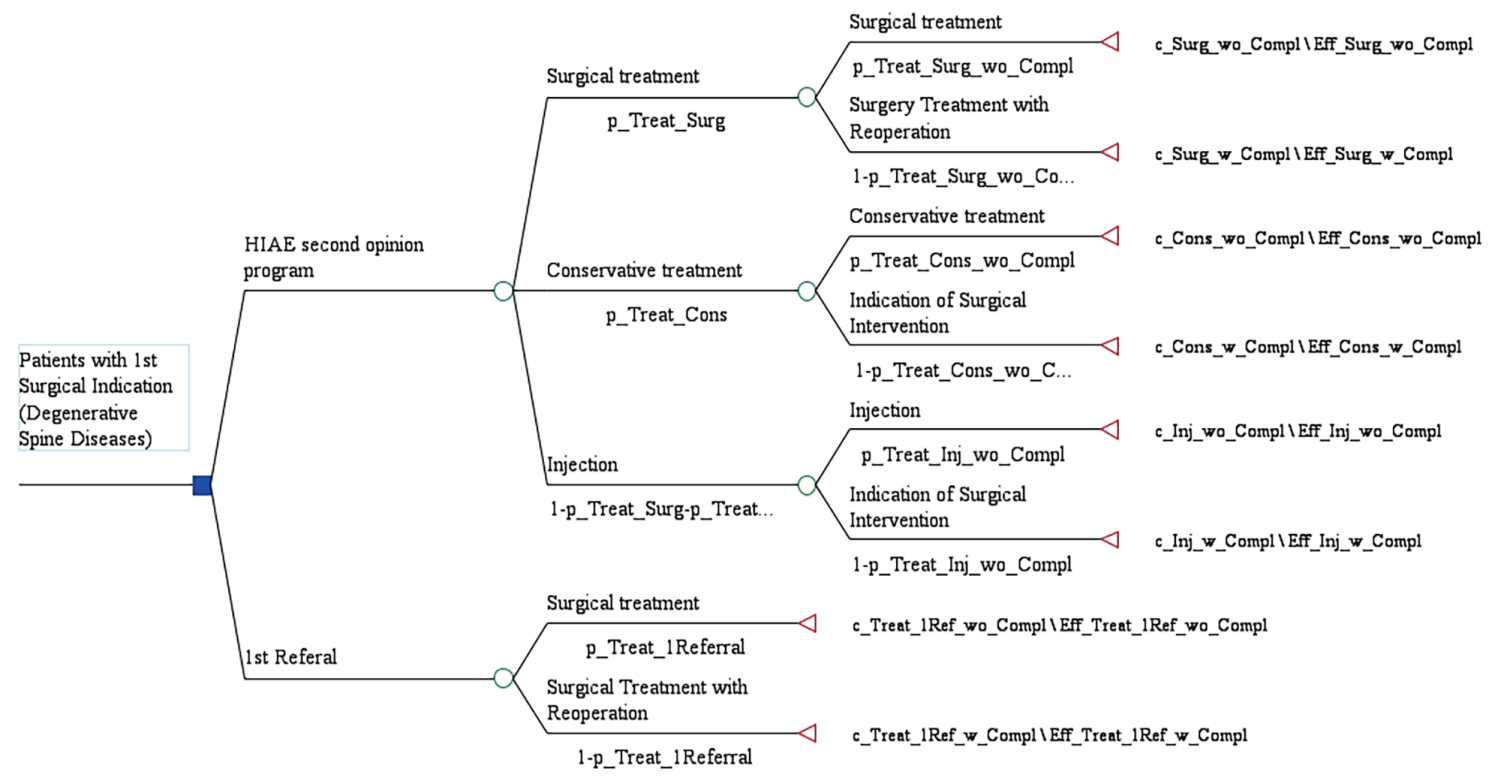
Decision-Tree Model with probabilities and costs. Measure of costs: Hospital costs and medical fees considering the month of June/2021. Outcome measure: Percentage of patients with clinical improvement measured as a difference in Utility between 2 years and baseline of at least the MIC

Figure 1 presents the structure of the decision tree considered in the analysis. The Incremental Cost Effectiveness Ratio (ICER) was calculated to describe the cost-effectiveness of the Second Opinion Program of Spine Surgery at the HIAE Spine Center.

The costs and probabilities considered in the economic modeling, as well as the confidence intervals and the Utility values obtained in each treatment group, are available in the supplementary file (Additional Table 2).

The ICER was calculated by dividing the difference in cost by the difference in the percentage of patients achieving the MIC according to the formula:

ICER = (Cost second opinion – Cost first referral) / (% MIC second opinion – % MIC first referral) [38].

The bootstrapping technique [40] was used to estimate the confidence interval of the incremental cost-effectiveness ratio. For the analysis, real hospital costs and outcomes data were used, representing the sample of patients collected, i.e., patients that realized the treatment recommended by second opinion. And, for the estimated data, the average costs and outcomes of surgical indication by First referral were used. Bootstrapping was performed with simulations of 1,000 samples randomly selected among the 1,088 patients included in the analysis, with replacement. 95% confidence intervals were calculated considering 2.5% and 97.5% percentiles.

For deterministic sensitivity analysis regarding probabilities, the lowest and highest values of the probability observed between 2011 and 2019 were used, segmented by year. And for the sensitivity analysis regarding hospital costs and outcomes, confidence intervals were calculated using real data from second indication and estimated for the initial indication of patients varying one standard deviation, the minimum value being the mean minus one standard deviation and the maximum value the mean plus one standard deviation.

To illustrate the sensitivity of the results, a tornado diagram was created. A tornado diagram is a visual representation commonly used in sensitivity analysis to evaluate how uncertainty in input parameters affects the outcomes of a cost-effectiveness analysis. It helps identify which parameters have the most significant influence on the outcomes and which contribute the most to the uncertainty in the analysis. For this purpose, parameters that affect the ICER, such as costs, effectiveness, and probabilities, were selected. Ranges were defined for each parameter to reflect the associated uncertainty or variability. This involved specifying the minimum and maximum values that each parameter could take. A sensitivity analysis was then conducted by varying each parameter within its defined range while keeping the other parameters constant. The ICER was recalculated for each variation.

R version 4.3.1 software was used for statistical tests and descriptive analysis (https://www.R-project.org/). The cost-effectiveness analysis constructed by the decision tree, the cost-effectiveness ratio graph, tornado diagram, and the sensitivity analysis were performed using the TreeAge Pro 2015 software (http://www.treeage.com).

**Fig. 2:**
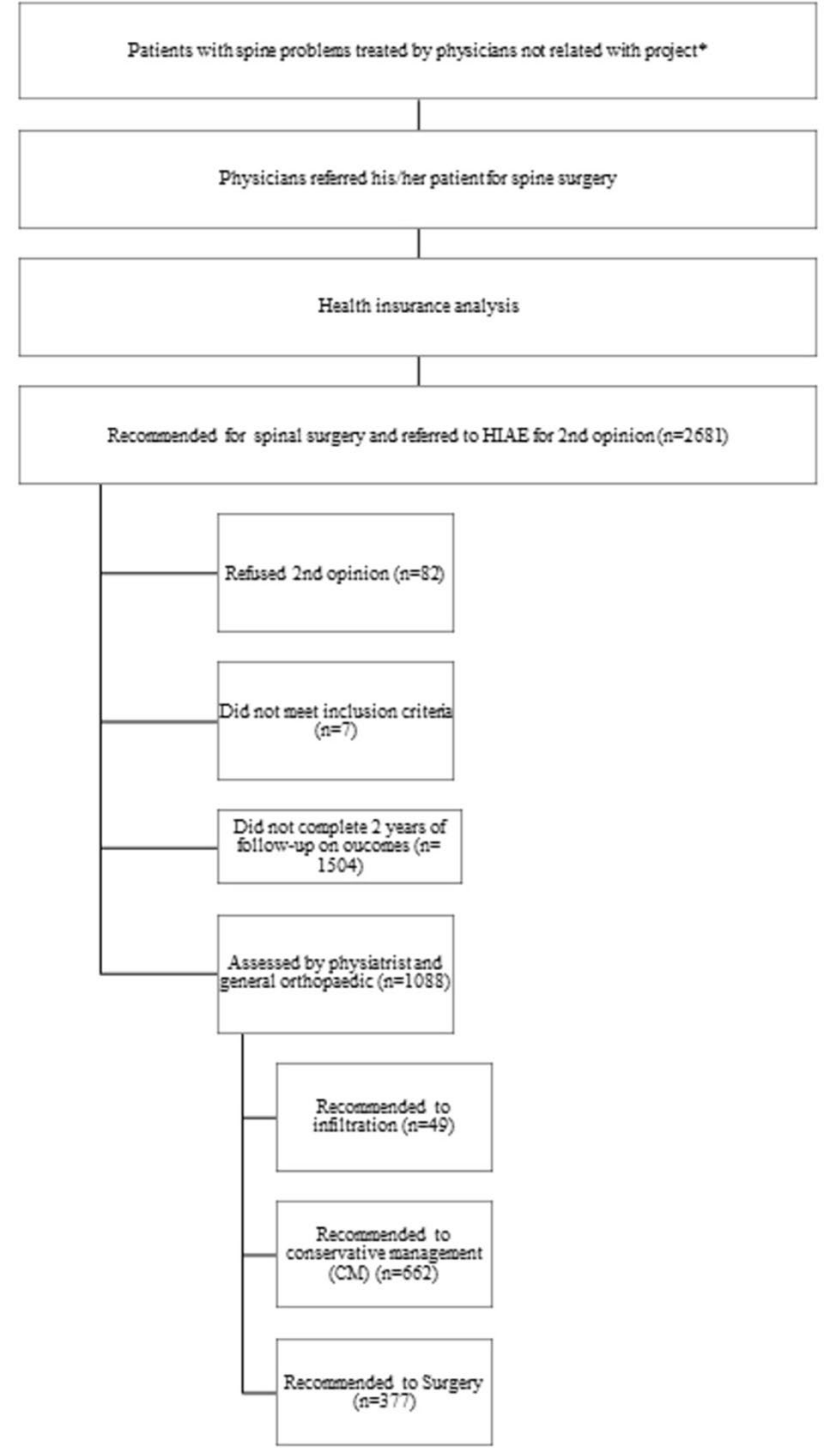
CONSORT diagram showing the selection of study participants based on inclusion and exclusion criteria, and the processes of selection and follow up

A CONSORT (Consolidated Standards of Reporting Trials) diagram explaining the participants’ selection and follow up processes is presented in Fig. 2. Economic analysis was performed with qualitative and quantitative characteristics assessed of 1,088 patients that agreed to the second opinion and completed 2 years of follow-up. These patients were categorized according to the treatment given after the second opinion program in injection, conservative treatment (physiotherapy) and surgery groups.

## Results

Of the 1,088 patients with a first referral for surgical treatment and completed 2-year follow-up, the second opinion program recommended injection treatment for 49 patients (4.5%); conservative management for 662 patients (60.8%) and surgical treatment for 377 patients (34.7%) (Fig. 2).

The first diagnoses and treatment indications made by the first referral and those made as part of the second opinion program are presented in the supplementary file (Additional Table 3).

Arthrodesis was the main treatment recommended by first referral (737 patients; 67.7%). Among these patients, with arthrodesis indication by first referral, the most frequently recommended treatment by the second opinion program was conservative management for 449 patients (60.9%), and different surgical approaches for 258 patients (35.0%), including arthrodesis, decompression, hernia and rhizotomy. In the overall analysis, the second opinion program recommended conservative management for most patients (662 patients; 60.8%). Regarding surgical indication by the second opinion program, the arthrodesis recommendation, a complex spine surgery, occurred in only 6.0% of cases. The percentage of agreement in the recommendation for arthrodesis was 3.7% between the second opinion program and the first referral. Furthermore, most surgical indications through the second opinion program were fewer complex surgeries, with decompression being recommended in 20.3% of the cases. (Table 2).

**Table 2:** Comparative treatment indication between first referral and second opinion program

		SECOND OPINION						
		Conservative	Injection	SURGICAL				TOTAL
				Arthrodesis	Decompression	Hernia	Rhizotomy	
**FIRST REFERRAL**	Arthrodesis	456 (41.9%)	21 (1.9%)	40 (3.7%)	213 (19.6%)	4 (0.4%)	3 (0.3%)	737(67.7%)
	Decompression	51 (4.7%)	4 (0.4%)		32 (2.9%)	8 (0.7%)		95 (8.7%)
	Hernia	93 (8.5%)	8 (0.7%)	20 (1.8%)	38 (3.5%)			159 (14.6%)
	Injection	43 (4.0%)	10 (0.9%)	3 (0.3%)	6 (0.6%)	2 (0.2%)		64 (5.9%)
	Rhizotomy	19 (1.7%)	6 (0.6%)	2 (0.2%)	5 (0.5%)	1 (0.1%)		33 (3.0%)
	TOTAL	662 (60.8%)	49 (4.5%)	65 (6.0%)	294 (27.0%)	15(1.4%)	3 (0.3%)	1088(100%)

The frequency of treatments recommended by second opinion for patients who completed the 2-year follow-up and for those who did not is described in the supplementary file (Additional Table 4), showing that the proportions of patients in each treatment, gender and age are similar in both groups.

Table 3 shows the characteristics of patients submitted to different treatments recommended by the second opinion program. Low back pain was more prevalent among patients, affecting 81.2% (n = 883), in contrast to cervical pain, diagnosed in only 18.8% of patients (n = 205). We did not observe differences between treatment groups regarding gender and age, suggesting that treatments were recommended irrespective of these characteristics. After 2 years, the percentage of patients who reached MIC was equivalent or significantly superior among patients who performed the treatment recommended by the second opinion program compared to the projected scenario if they had performed the treatment recommended by the first referral (*p* < 0.01), regardless of the treatment performed (79.3% surgical; 70.1% conservative and 75.5% injection). To compare the rate of patients who reached MIC between the second opinion program and the first referral, data from patients with the same diagnosis, characteristics and who underwent the surgery recommended by the first referral at HIAE were used, that is, clinical data of the patients surgically treated at the hospital, thus estimating the percentage of patients achieving the MIC if they had been submitted to the first referral treatment, as described in the Methods section. The results, based in MIC analysis, show that the rate of patients who supposedly would have improved with surgery recommended by the first referral is 80.6%, comparable to the rate observed after surgical treatment by the second opinion program, which was 79.3%.

**Table 3:** Profile of patients treated by the second opinion program in a 2-year follow-up and rate of patients that achieve MIC and estimate MIC

		SURGICAL	CONSERVATIVE	INJECTION	*p*-value
		(n = 377)	(n = 662)	(n = 49)	
LOW BACK / CERVICAL	LOW BACK	317(84.1%)	518(78.2%)	48(98.0%)	< 0.01
	CERVICAL	60(15.9%)	144(21.8%)	1 (2.0%)	
GENDER	FEMALE	187(49.6%)	357(53.9%)	21(44.9%)	0.2482
	MALE	190(49.6%)	305(46.1%)	28(57.1%)	
AGE AVERAGE (SD)		46.6(13.8)	45.9(12.2)	46.5(11.4)	0.8798
MIC 2nd OPINION	YES	299(79.3%)	464(70.1%)	37(75.5%)	< 0.01
	NO	78(20.7%)	198(29.9%)	12(24.5%)	
ESTIMATED MIC (FIRST REFERRAL)	YES	304(80.6%)	385(58.2%)	27(55.1%)	< 0.01
	NO	73(19.4%)	277(41.8%)	22(44.9%)	

The mean Utility score at baseline was different between patients recommended to surgery and those recommended to conservative or injection treatment (0.44 vs. 0.57 and 0.55 respectively) by the second opinion program, suggesting that patients undergoing surgery had a worse quality of life at baseline. After 2 years of treatment, the mean Utility increased for all patients, reaching similar scores in different treatments. This result indicates that the treatment recommended by the second opinion was adequate for the patients to improve their quality of life. Also, the average Utility score achieved by the second opinion program was comparable to the Utility estimated for the first referral (Table 4).

**Table 4:** Comparison of mean Utility scores at baseline and after 2 years of treatment by second opinion versus first referral

	SECOND OPINION (Utility)			FIRST REFERRAL (Utility Estimated)		
	Baseline	2 years	*p*-value	Baseline	2 years	*p*-value
SURGICAL	0.44(0.18)	0.76(0.22)	< 0.01	0.44(0.18)	0.77(0.08)	< 0.01
CONSERVATIVE	0.57(0.15)	0.79(0.21)	< 0.01	0.57(0.15)	0.76(0.09)	< 0.01
INJECTION	0.55(0.17)	0.80(0.23)	< 0.01	0.55(0.17)	0.73(0.12)	< 0.01

The costs and probabilities considered in the economic modeling, as well as the Utility values obtained in the respective treatment groups, are presented in Additional Tables 2 and 5. Regarding conservative treatment, it was observed that only 5% of patients required surgical intervention within 2 years after treatment, resulting in a cost increase of $16,876.98 (from $1,1,628.40 to $18,505.38). While the cost of initial surgical treatment was $6,328.36, with a 9% reoperation rate and $15,659.78 additional cost in case a reintervention was needed. (Additional Table 2).

Table 5 shows the results of the base-case analysis. In this analysis, second opinion treatment is the dominant strategy, demonstrated by its greater effectiveness in relation to the percentage of patients with improvement in 2 years greater than MIC and lower average costs. The financial savings generated by the second opinion program are due to the indication of conservative treatment and lower complexity surgeries in surgical cases. The second opinion resulted in better results compared to the estimated data for patients from the first referral, if they had performed the proposed surgery in the same hospital. According to the model presented in the perspective of a tertiary level private hospital second, opinion strategy could result in financial savings and benefits for patients.

**Table 5:** Incremental cost-effectiveness ratio between comparators: HIAE second opinion program versus first referral

Strategy	Cost (US$)	Effectiveness (% patients achieving MIC) *	Incremental Cost	Incremental Effectiveness	ICER (Incremental Cost / Incremental Effectiveness)
HIAE Second Opinion Program	$ 4,115.19	73.5%	-$6,705.40	7.7%	-$ 87,066.19
First referral	$ 10,820.59	65.8%			

* Effectiveness (percentage of patients with improvement in 2 years greater than reference MIC)

ICER = Incremental cost-effectiveness ratio

**Fig. 3:**
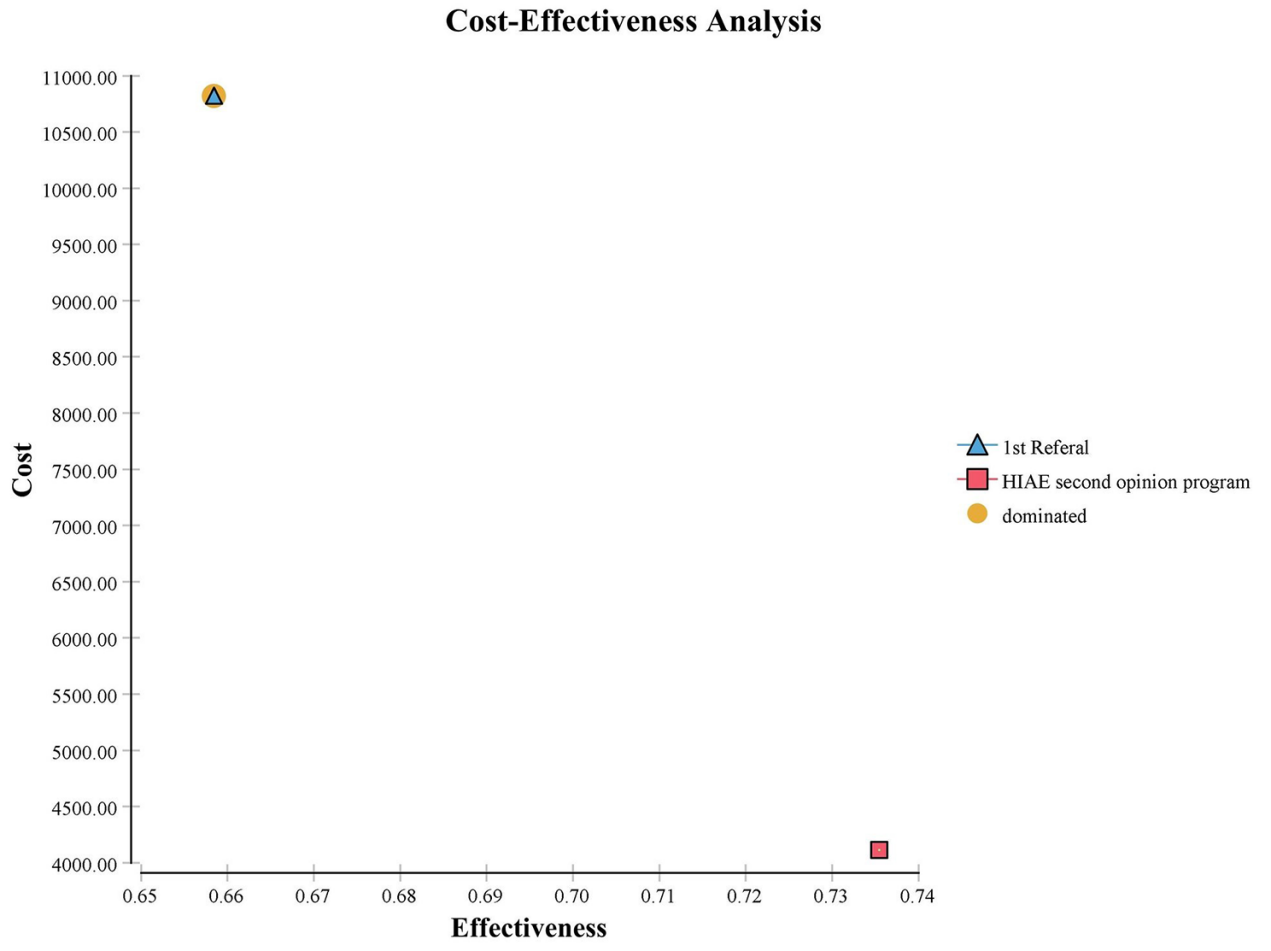
Cost-effectiveness plane for HIAE second opinion (dominant strategy) program versus First referral (dominated strategy)

Figure 3 illustrates the cost-effectiveness plane for HIAE second opinion program versus First referral. An incremental effectiveness result of 7.7% was obtained when comparing the percentage of patients who reached the MIC between the first indication and the second indication and a negative incremental cost, that is, a saving of -$6,705.40 when comparing the second opinion strategy versus the first indication, resulting in an ICER of -$87,066.19. That is, for each additional patient that reached the MIC, in comparison with the first indication, there is a saving of $87,066.19. For the study, with 1,088 patients included in the analysis, resulting in a total savings of $7,295,475.20, considering the ICER result, an improvement in effectiveness of 7.7% represents about 84 patients who at the first indication would not have a significant improvement and, with the second opinion program, they start to have this improvement.

**Fig. 4:**
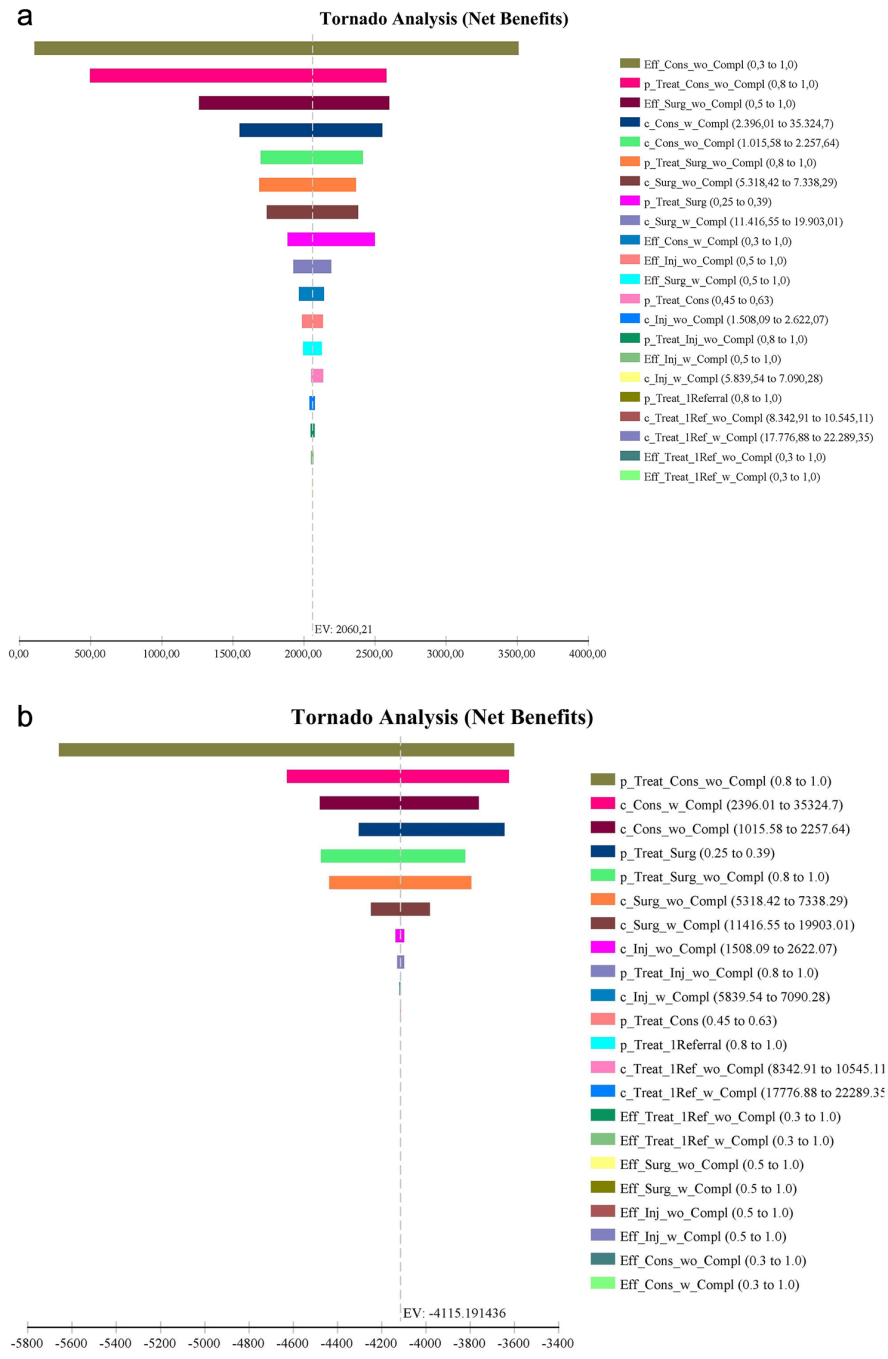
Tornado diagram of the HIAE second opinion program versus First referral. Indicating the variables in descending order of influence on (**a**) Variables in descending order of influence on the Net Benefits considering a willingness to pay of 1 per capita GDP and (**b**) on the Net Benefits considering a willingness to pay of 0 (1 per capita is US$ 8.396.94)

The tornado diagram (Fig. 4a and b) shows that the ICER is more sensitive to changes in the cost of conservative management that evolves with complications within 2 years and requires surgical intervention. The parameters of costs, effectiveness, and probabilities were ranked according to their impact on the ICER. This was done by comparing the range of ICER values associated with variations in each parameter. The parameters were listed on the vertical axis, while the corresponding ranges of ICER values were represented by horizontal bars. The length of each bar indicates how much the parameter affects the ICER.

The probability of indication of patients for surgical treatment in the second opinion is also a parameter that impacts the results and was varied, taking into account the probabilities of indication of patients during the years of 2011 to 2019, where the probability of surgical indication ranged from 25 to 39%, values simulated in the deterministic sensitivity analysis.

The deterministic sensitivity analysis [41] showed that none of the variations led to a scenario in which the results are not cost saving in favor of the second opinion program.

**Fig. 5:**
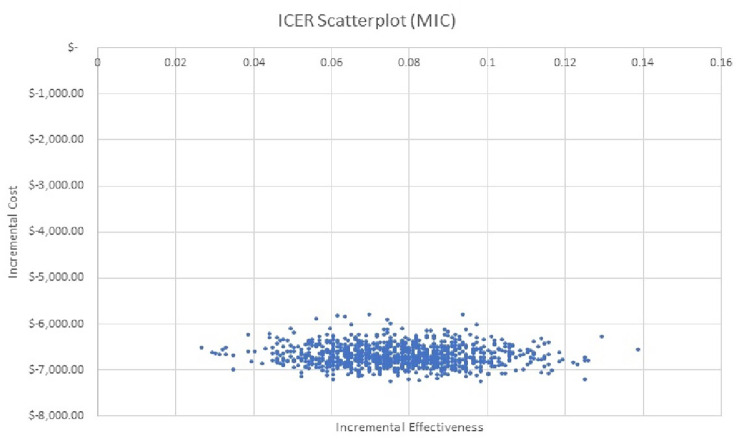
Cost-effectiveness Scatterplot. The empirical sample distribution of ICER was based on 1000 simulated results

A probabilistic sensitivity analysis was performed to estimate a confidence interval for the ICER using the bootstrapping methodology [40]. The bootstrapping analysis used 1,000 random simulations, replacing the data from the 1,088 patients analyzed, obtaining, at the end of the simulations, the empirical sampling distribution of the ICER (Fig. 5).

The incremental cost-effectiveness ratio uncertainty, assessed by the bootstrapping method, found that the second opinion program savings could range from -$7,291.20 to -$5,860.78 per patient, in addition to an increase in effectiveness that ranged between 0.0276 and 0.1333. Even if the indication of surgery increases up to 60% after the second opinion, the program remains cost-effective. These results confirm the robustness of the analysis in favor of the second opinion program being cost saving while also improving patient outcomes.

## Discussion

The present study aimed to evaluate the expenditures and clinical outcomes of the second opinion on spine surgeries program compared to those treated outside of the program. For comparison analysis, we used data based on our clinical practice, using data from patients who underwent the same type of surgical procedure as recommended by the first referral. We proposed a new strategy to measure cost-effectiveness, using as clinical endpoint, the MIC in the quality of life reported by patients, including the satisfaction questionnaire regarding the treatment.

After total evaluation of 1,088 patients, 60.8% of the patients received conservative management; 4.5% injection treatment and 34.7% surgical treatment. Considering that all patients had prior surgical indication, this result confirms the absence of consensus in the management and treatment options in degenerative spine care. Reasons raised to justify such disparity include but are not limited to different initial diagnosis, inadequate clinical radiologic correlation, and/or lack of adherence to clinical guidelines which recommend initial conservative management [42, 43].

Among the 34.7% of patients referred to surgery after second opinion, there were discrepancies regarding the type of surgery when compared to the first referral. Regarding surgery for lumbar degenerative diseases, 737 arthrodesis had been previously recommended against only 65 after second opinion evaluation. Absence of consensus and the variety and complexity of existing surgery types to treat degenerative spine diseases underlie the results described in the current literature [11, 12, 14].

There is a lack of clearly defined reasons that can explain the discrepancy among surgical indications to treat degenerative neck or low back diseases [31, 32]. Some authors imply that different factors contribute to the increased frequency of complex surgery indications. These factors include population characteristics, innovative technology with new techniques and instruments, improvement of analgesic techniques and supportive care, new implant types, financial incentives to hospitals and surgeons to perform more complex procedures, lack of consensus of treatments based on scientific studies analyzing the pros and cons of such techniques, and the support from the implant industry [33, 44].

Most patients were initially evaluated by surgical specialists, neurosurgeons and orthopedists, who generally tend to provide more surgical care [45] which, by nature, is more expensive and only after a few weeks patients were evaluated by the second opinion program. This leads to the conclusion that the timing of medical evaluation may also explain the discrepancy in medical decision, considering that the degenerative aspect of the diseases, patient complaints and severity of symptoms could have been different from the first opinion [31, 32].

Program process flow and the decision-making strategy from a second opinion spine program is one of the core aspects of this model. Patients are evaluated and re-evaluated by different specialists and, when there is a recommendation for surgery, the surgery follows a protocol agreed upon by the multidisciplinary team during the weekly board review.

The second opinion program proved to be dominant generating savings of $87,066.19 per additional patient with improvement in 2 years greater than reference MIC, based on the patient perception about the improvement in their quality of life. Part of this result is due to the potential that the second opinion program has to reduce unnecessary care, which generates lower costs for the paying source (insurers, in most cases) and also increase on the referrals to conservative management and less complex surgical procedures.

Quality of life measures are used to evaluate the efficacy of treatment modalities in any disease including the SRD’s [38, 46, 47]. However, a recent study showed the difficulty to establish a universal value that represents a MIC in the quality of life of the patients with SRD after different treatments [48]. The MIC is related to the smallest change in score of EQ-5D-3 L that patients consider important, using the anchor-based method [38]. So, for this study the effectiveness of the second opinion program was calculated as the percentage of patients considered as responders, for having achieved the MIC in 2 years, based on the difference in quality of life (Utility). This approach considered the patient opinion about their own clinical improvement.

Effectiveness of conservative management provided could be questioned since patients have received a spine surgery referral from the first referral. However, after 2-years of follow-up the results demonstrate that the quality of life of patients submitted to this treatment improved, confirming that the treatment was effective. Within this period of follow-up only 5.2% of patients (N = 31/600) required revision of treatment and were submitted to surgery, although even on these cases, the Utility improved on follow-up, confirming the cost-effectiveness in those patients.

Economic evaluations are considered important by many health care providers, payers and patients. It may be particularly important when comparing interventions that provide similar clinical effectiveness. A recent systematic review concerning economic evaluations of exercise therapy in the treatment of back pain pointed out as a future direction for this line of research the comparison between exercise therapy with drugs and surgery, since most health economic studies regarding spine procedures are linked to technique and/or instruments used [27].

Moreover, in such circumstances it is imperative to establish new methods of medical evaluation and multi-disciplinary decision-making. Additionally, these protocols must be evidence based to achieve sustainability within the health care system [28]. In this manner, integrated work by healthcare professionals and health management teams is essential. This project provides a demonstration of an innovative, collaborative model that is both costs saving and sustainable [3, 5, 19, 49].

The model of evaluation conducted on the tertiary hospital (HIAE) followed fundamental precepts and important current trends in health care. As suggested by Porter et al., reforms in healthcare should create value for patients by creating iterative, evaluative cycles to improve quality of care and medical practices should be organized around medical conditions and care cycles rather than around specialties or procedures. The authors hypothesize that the use of these “integrated practice units” will result in improved professional satisfaction and increased value for patients and payers [50].

Unless physicians improve health and health care value for patients, they will inevitably face ever-increasing administrative control of medicine [50]. This project was designed in accordance to those principles and assurance that ethics in patient care are respected.

According to the Institute of Healthcare Improvement (IHI), “there is a lack of resources focused on reducing healthcare costs, though a correct allocation and focus on the patient experience can lead to quality improvement of healthcare provided, avoiding unnecessary treatment and driving cost optimization to other areas in need” [51, 52]. Also, current viewpoints recommend the development and implementation of cost-effective strategies that provide access to effective care in low-income and middle-income countries [53].

The HIAE second opinion program is a viable option to augment and potentially change current clinical practice. Our data confirms this trend showing improved patient outcomes while simultaneously providing savings for limited healthcare resources.

The study has some limitations. Firstly, treatment costs and outcomes of the first referral were inferred using the data from patients who underwent the same type of surgical procedure at HIAE as recommended by the first referral and with a 2-year follow-up. Thus, this estimate was performed based on our clinical practice. However, the procedure costs vary widely according to procedure type and quantity of materials and implants used in each hospital. Secondly, the standardization of materials and equipment used by the medical team allowed optimization of costs and expenses. This factor could have contributed to a better cost effectiveness in this study. To avoid inflationary discrepancies in the period analyzed, hospital costs and medical fees were analyzed based on the values negotiated in June 2021, creating a simulated scenario where all patients had cost behavior for the year 2021. Thirdly, this study conducted at a single center, and therefore the findings may not be applicable to other institutions. However, it is important to mention that our hospital is open to external surgeons with different backgrounds, surgical techniques, and levels of experience. Fourthly, the patients referred to HIAE may introduce bias, but we did not control for this as the decision to seek a second opinion on spine-related matters is determined by the health insurance center. Despite these limitations, it is understood that this study is relevant to support decision-making in health, considering a scenario in which the patient’s quality of life is positively impacted through the MIC and, in addition, financial savings are generated, favoring the sustainability of the healthcare system.

Although there are limitations, it is important to note that this study is relevant for informing health-related decision-making. It considers a scenario where the patient’s quality of life is improved through the use of MIC, while also generating financial savings that contribute to the sustainability of the healthcare system.

## Conclusions

This study demonstrates that the second opinion program for patients with SRD was cost saving when compared to the first referral, from the perspective of the hospital, based on real life data.

This program should be considered in clinical settings as an alternative to immediate referral for spinal fusion as our data show the potential for better allocation of resources and improved healthcare quality.

### Electronic supplementary material

Below is the link to the electronic supplementary material.

**Supplementary Material 1: Additional Table 1**. Utility data at baseline and 2 years after intervention and patient satisfaction with treatment outcome used to calculate minimal important change (MIC)

**Supplementary Material 2: Addtional Table 2**. Values of parameters used in the model and sensitivity analysis

**Supplementary Material 3: Additional Table 3**. Comparison of treatment indications by the first referral versus the second opinion program

**Supplementary Material 4: Additional Table 4**. Profile of patients treated in the second opinion program

**Supplementary Material 5:** Database of economic analysis. Qualitative and quantitative characteristics of 1,088 patients that agreed to the second opinion and completed 2 years of follow-up

## Data Availability

All data generated and analyzed during this study are included in supplementary information files.
